# Dataset of emission and excitation spectra, UV–vis absorption spectra, and XPS spectra of graphitic C_3_N_4_

**DOI:** 10.1016/j.dib.2018.09.123

**Published:** 2018-10-03

**Authors:** Liangrui He, Mi Fei, Jie Chen, Yunfei Tian, Yang Jiang, Yang Huang, Kai Xu, Juntao Hu, Zhi Zhao, Qiuhong Zhang, Haiyong Ni, Lei Chen

**Affiliations:** a School of Materials Science and Engineering, Hefei University of Technology, Hefei 230009, China; b National Engineering Lab of Special Display Technology, State Key Lab of Advanced Display Technology, Academy of Opto-Electronic Technology, Hefei University of Technology, Hefei 230009, China; c Hefei National Laboratory for Physical Sciences at the Microscale, University of Science and Technology of China, Hefei 230026, China; d Guangdong Province Key Laboratory of Rare Earth Development and Application, Guangdong Research Institute of Rare Metals, Guangdong Academy of Sciences, Guangzhou 510651, China; e Intelligent Manufacturing Institute of Hefei University of Technology, Hefei 230051, China

**Keywords:** Graphitic C_3_N_4_, Luminescence spectra, Absorption spectra, X-ray photoelectron spectroscopy (XPS)

## Abstract

In this data article, the normalized emission and excitation spectra, the ultraviolet-visible (UV–vis) absorption spectra, and the X-ray photoelectron spectroscopy (XPS) of bulk-powders and nano-structured graphitic C_3_N_4_ (g-C_3_N_4_) were presented, which are helpful to get insight into the crystal and electronic structures of g-C_3_N_4_, especially on determining the energy levels and the mechanisms of luminescence originating from electron transitions. This data article is related to our recent publication (He et al., in press) [1]. The absorption, excitation and emission spectra are vital to illustrate the optoelectronic performances in terms of photoluminescence, photocatalysis, electroluminescence, etc., from the viewpoint of electron transitions intrinsically.

**Specifications table**

**Table:** 

Subject area	Physics, chemistry, materials science
More specific subject area	Solid-state luminescence in condensed luminescence, solid-state structure in inorganic chemistry, functional materials in photoluminescence, electroluminescence, photocatalyst, etc.
Type of data	figure
How data was acquired	Fluorescence spectrophotometer (F-4600 Hitachi), ultraviolet-visible (UV-VIS) spectroscopy (UV-3600, Shimadzu), X-ray Photoelectron Spectrometer (ESCALAB250Xi, Thermo)
Data format	Raw, analyzed
Experimental factors	Temperatures, reaction atmospheres, bulk-powder or nano-structure
Experimental features	The intensity and wavelength of emission, excitation, and absorption spectra; and the binding energy position and the counts per second in XPS
Data source location	Hefei, China
Data accessibility	Data are provided with this article

**Value of the data**

• The electron transitions and their corresponding energy levels could be identified from the normalized emission and excitation spectra.
• The fast relaxation of electrons from excited states to the ground state without the Stokes shift radiation could be discriminated by comparing absorption spectra with emission spectra together.
• The XPS data are helpful to reason out the way and reaction process of the thermal condensation of melamine to form g-C_3_N_4_ and thereby, to illustrate the performances from viewpoint of crystal structure.
• The emission, excitation and absorption spectra of g-C_3_N_4_ are helpful to recognize its electronic structure.
• The electronic and crystal structures are useful to interpret the intrinsic properties of g-C_3_N_4_ in terms of photoluminescence, electroluminescence, photocatalysis, etc.

## 1 Data

**Fig. 1:**
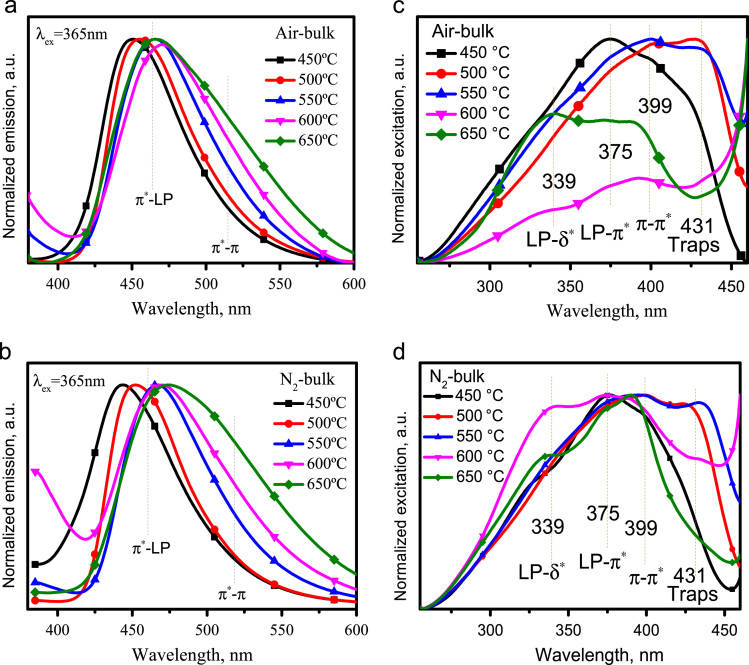
The normalized emission and excitation spectra of bulk powders synthesized at different temperatures in Air and N_2_ atmospheres: a, b, the emission spectra under excitation of 365 nm; c, d, the excitation spectra achieved by monitoring the strongest emission in a,b.

The normalized emission spectra of g-C_3_N_4_ powders synthesized at various temperatures in air and N_2_ atmospheres are shown in Fig. 1a,b, respectively, which shows that the emission peaks redshift with temperature increasing from 450 to 600 °C. The asymmetrical emission band mainly consists of the π*–Lp (lone pair electrons) and π*–π transitions. The normalized excitation spectra of g-C_3_N_4_ powders synthesized at various temperatures in air and N_2_ atmospheres are displayed in as Fig. 1c,d, in which the excitation peaks at 339, 375, 399 and 431 nm were attributed to the LP–δ* (* indicates the antibond), LP–π*, π–π*, and traps absorption, respectively.

**Fig. 2:**
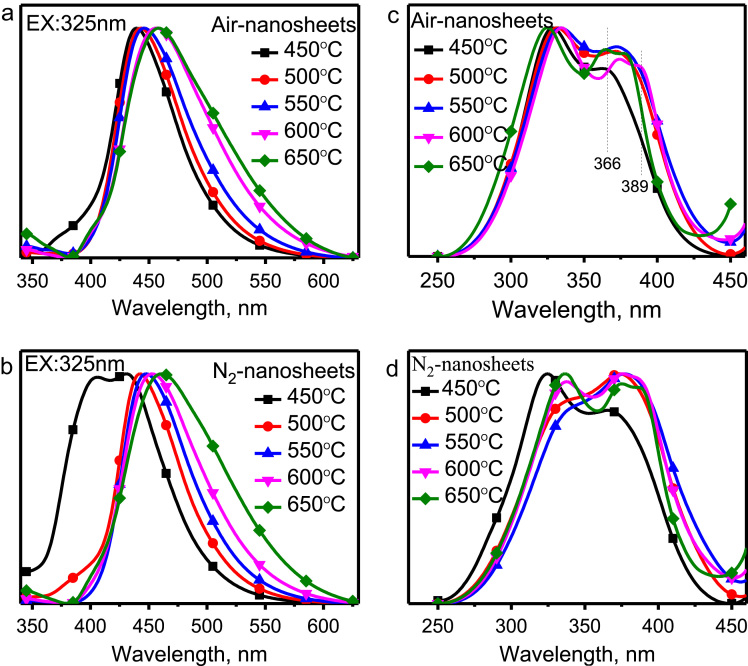
The normalized emission and excitation spectra of quantum dots achieved by ultrasonic exfoliating the above bulk powders synthesized at different temperatures in Air and N_2_ atmospheres, respectively; a, b, the emission excited with 325 nm; c, d, the excitation spectra synthesized in N_2_, respectively.

The normalized emission and excitation spectra of g-C_3_N_4_ quantum dots (QDs) are presented in Fig. 2. After being exfoliated into QDs, the emission and excitation peaks shift towards high energy direction (i.e., blueshift) evidently, as seen by the comparison of Fig. 2a–d with Fig. 1a–d. Besides, the δ*–Lp transition peaked at 405 nm was observed in Fig. 2b for the sample synthesized at 450 °C under N_2_ atmosphere, suggesting the electrons cannot relax from the high-energy δ* state to the low-energy π* state efficiently. Maybe, the π orbital is not well formed under the condition of 450 °C in N_2_ atmosphere.

**Fig. 3:**
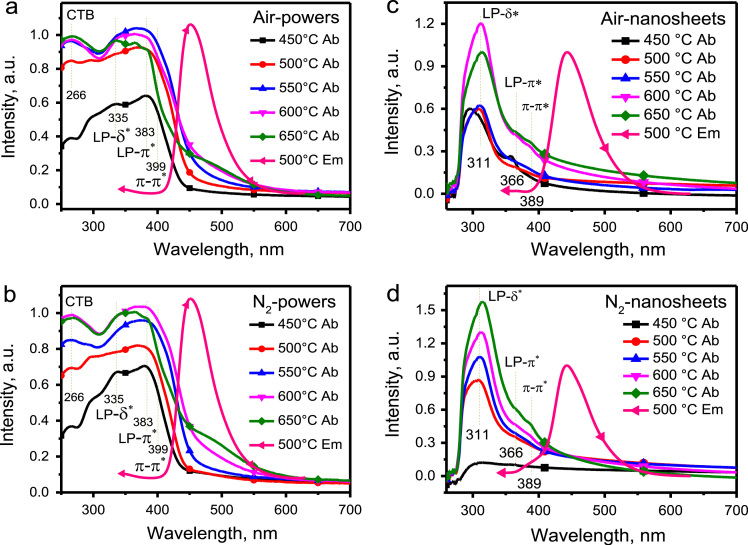
The comparison of absorption spectra with emission spectra for the bulk powders and quantum dots: a,b, the bulk powders synthesized at variant temperatures in Air and N_2_ atmospheres, respectively; c,d, the quantum dots obtained by ultrasonic exfoliating the bulk powders in a,b.

The absorption spectra of g-C_3_N_4_ bulk powders synthesized at various temperatures in air and N_2_ atmospheres, respectively, are shown in Fig. 3. Besides the LP–δ*, LP–π*, and π–π*, transitions as correspond to the excitation bands in Fig. 2a, b, one band peaked at about 266 nm was observed in Fig. 3a, b, which was attributed to the charge effect band (i.e., photocurrent, marked with CTB). The CTB was nearly not observed under 450 °C. However, the increase of CTB in intensity with an increase of temperature from 500 to 650 °C suggests that the exorbitant condensation of the g-C_3_N_4_ easily result in the photocurrent, which can explain the decrease of g-C_3_N_4_ luminescence upon increasing temperature from 500 to 650 °C. Moreover, the absorption within 440–600 nm increases with temperature increasing from 450 to 650 °C. The position of this absorption band is consistent with the absorption wavelength of traps in Fig. 1c, d. So, it is naturally to assign the absorption band within 440–600 nm to crystal defects. Besides, the absorption within 440–600 nm overlaps with the emission band very well, as comparison of absorption spectra with emission spectrum displayed in Fig. 3a, b. Therefore, the absorption within 440–600 nm is mainly caused by the fast relaxation of electrons from the high-energy excited states to the ground band without the Stokes shift, overlaying with the absorption of crystal traps. After being exfoliated into QDs, the absorption bands of CTB and fast relaxation disappear, as seen from Fig. 3c, d.

The intensity of the LP–π*, and π–π* absorptions of g-C_3_N_4_ QDs in Fig. 3c, d are far weaker over those of bulk powders in Fig. 3a, b, suggesting the π orbital was partially damaged or broken during the process of ultrasonic exfoliating and in turn resulting in electrons cannot relax from high-energy δ* to low-energy π* state efficiently. Accordingly, the LP-δ* transition dominates the absorption of g-C_3_N_4_ QDs in Fig. 3c, d.

**Fig. 4:**
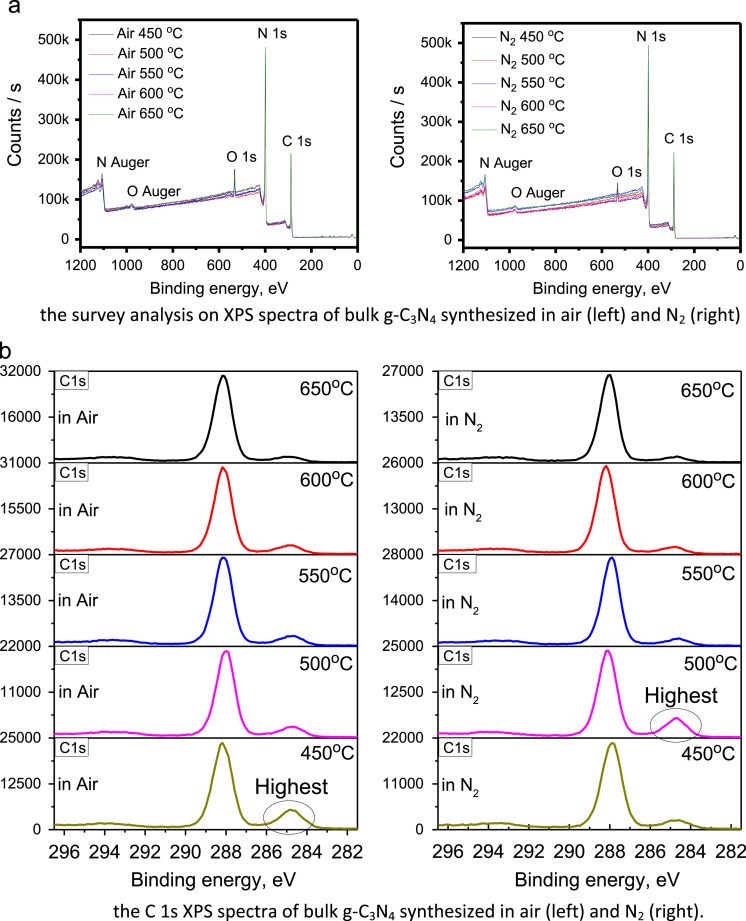

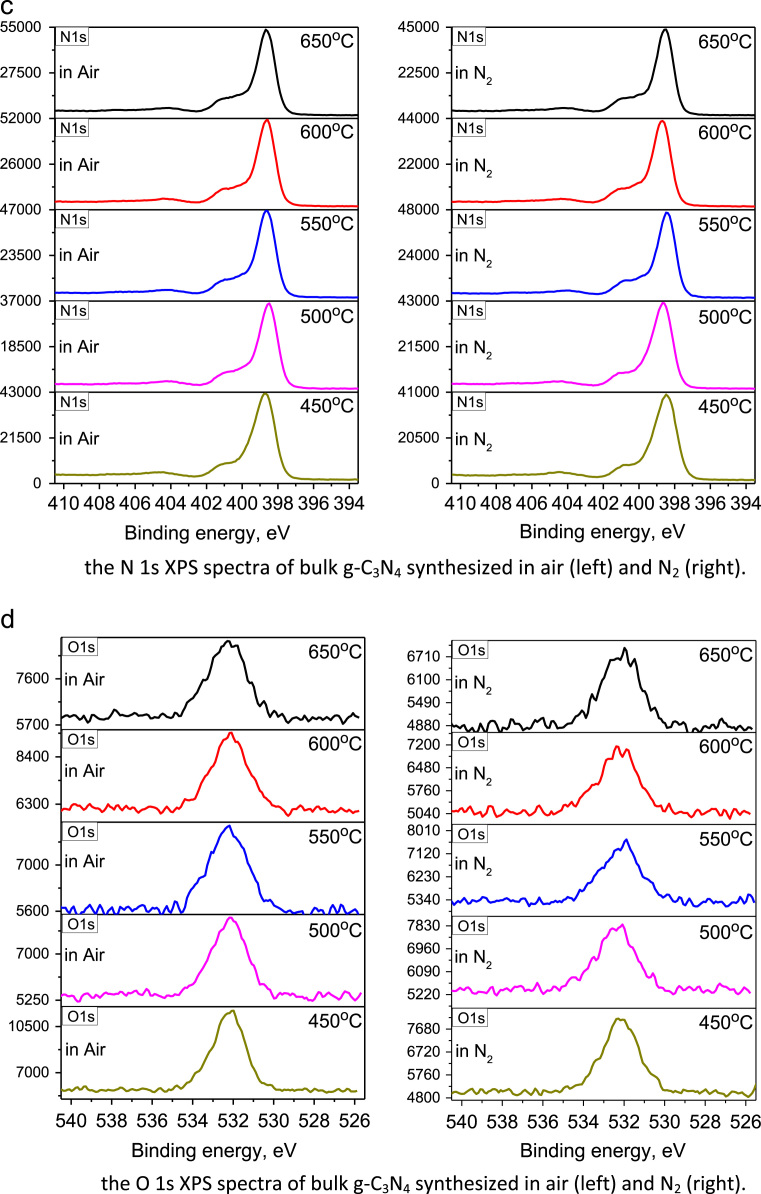
The survey, C 1s, N 1s, and O 1s XPS spectra of bulk g-C_3_N_4_ powders synthesized at variant temperatures in air and N_2_, respectively.

**Fig. 5:**
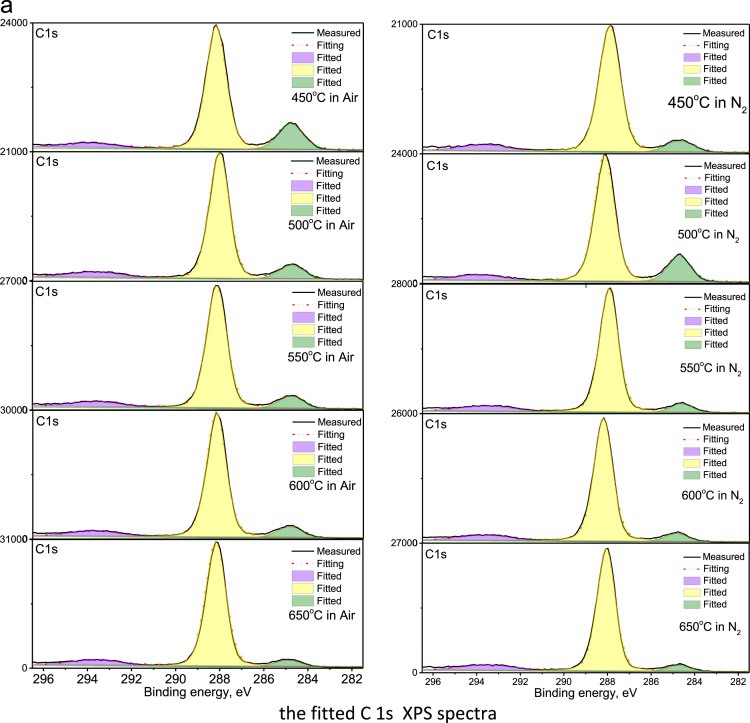

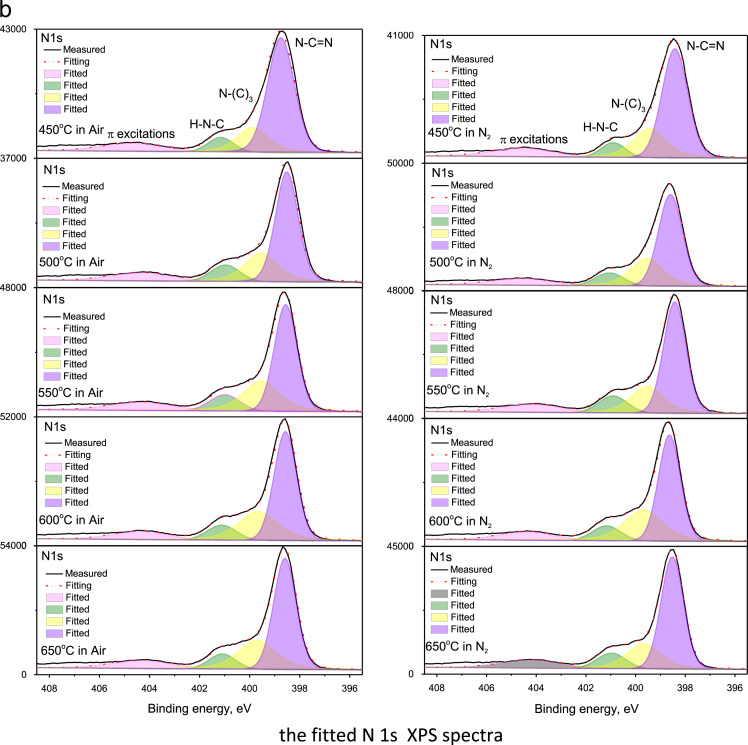
The fitted C 1s and N 1s XPS spectra, as corresponding to Fig. 4b,c, by using the XPS PEAK 4.1 program, of g-C_3_N_4_ powders synthesized at variant temperatures in air and N_2_, respectively.

The raw C 1s and N 1s XPS of g-C_3_N_4_ bulk powders and their fitted spectra by using the XPS PEAK 4.1 program are depicted in Fig. 4 and Fig. 5, respectively. The composition of C, N and O elements for the samples synthesized at various temperature in air and N_2_ atmospheres was summarized in Table 1. The analyses on XPS spectra in Fig. 4, Fig. 5 confirm that the structure of g-C_3_N_4_ consists of basic unit of tri-s-triazine ring, which is connected by the N atoms to form a π–conjugated polymeric network [2], [3], [4], [5], [6], [7], [8].

**Table 1:** the percent of C, N and O atoms in bulk g-C_3_N_4_ powders obtained by fitting the XPS spectra in Fig. 4 by using the XPS PEAK 4.1 program, as corresponding to Fig. 5.

**Items**	**450 °C**	**500 °C**	**550 °C**	**600 °C**	**650 °C**	**Conditions**
C1s	42.04	42.91	42.14	42.12	41.42	Synthesized in Air
N1s	51.8	52.62	54.01	53.63	54.58	
O1s	6.16	4.47	3.85	4.26	4	
C1s	39.44	43.55	41.81	41.91	41.83	Synthesized in N_2_
N1s	55.95	52.78	54.68	54.66	54.45	
O1s	4.61	3.68	3.51	3.43	3.72	

Moreover, the highest ratio of graphitic-to-triazine carbon was observed in the sample synthesized at 500 °C in N_2_ atmosphere and the second highest ratio of graphitic-to-triazine carbon was observed in the sample synthesized at 450 °C in air ambient. These ratios are consistent with the strongest and the second strongest luminescence of g-C_3_N_4_ powders presented in Fig. 2 and Fig. 3, respectively, in Ref. [1], indicating the luminescence efficiency is related with the type of carbon existence closely.

## 2 Experimental design, materials and methods

The material and methods used to obtain the data of emission and excitation, absorption, and XPS spectra were described in [1]. The emission and excitation spectra of bulk-powders and nano-structured g-C_3_N_4_ were collected with Hitachi F4600 spectrometer, and the spectra in Fig. 1, Fig. 2 were normalized to determine the energy levels. The absorption spectra in Fig. 3, recorded with UV-3600 spectra, were further in comparison with emission spectra to determine electron transition and the mechanisms thereof. The original XPS spectra, including the survey, C 1s, N 1s, and O 1s, of bulk g-C_3_N_4_ powders, measured with using the Thermo ESCALAB250Xi X-ray Photoelectron Spectrometer, were displayed in Fig. 4. The C 1s and N 1s XPS were fitted by using the XPS PEAK 4.1 program, as shown in Fig. 5, to reveal the way of thermal condensation and chemical bonding.
