# Supplementary material for: Dataset of emission and excitation spectra, UV–vis absorption spectra, and XPS spectra of graphitic C3N4
Source: Data Brief. 2018 Oct 3;21:501–10. doi: 10.1016/j.dib.2018.09.123 (PMC6199237; doi:10.1016/j.dib.2018.09.123)
Supplement: Supplementary file 1 — Transparency document [file mmc1.pdf]

Conflicts of Interest Statement

Manuscript title:

Dataset of emission and excitation spectra, UV-VIS absorption spectra, and XPS spectra of graphitic C3N4

The authors whose names are listed immediately below certify that they have NO affiliations with or involvement in any organization or entity with any financial interest (such as honoraria; educational grants; participation in speakers' bureaus; membership, employment, consultancies, stock ownership, or other equity interest; and expert testimony or patent-licensing arrangements), or non-financial interest (such as personal or professional relationships, affiliations, knowledge or beliefs) in the subject matter or materials discussed in this manuscript.

Author names:

Liangrui He1,Mi Fei1, Jie Chen, Yunfei Tian, Yang Jiang, Yang Huang, Kai Xu, Juntao Hu, Zhi Zhao, Qiuhong Zhang, Haiyong Ni, Lei Chen

The authors whose names are listed immediately below report the following details of affiliation or involvement in an organization or entity with a financial or non-financial interest in the subject matter or materials discussed in this manuscript. Please specify the nature of the conflict on a separate sheet of paper if the space below is inadequate.

Author names:

Liangrui He Mi Fei,Jie Chen, Yunfei Tian, Yang Jiang, Lei Chen
School of Materials Science and Engineering, Hefei University of Technology, Hefei 230009, China

Yang Huang, Kai Xu, Juntao Hu
National Engineering Lab of Special Display Technology, State Key Lab of Advanced Display Technology, Academy of Opto-Electronic Technology, Hefei University of Technology, Hefei 230009, China.

Zhi Zhao
Hefei National Laboratory for Physical Sciences at the Microscale, University of Science and Technology of China, Hefei 230026, China

Qiuhong Zhang, Haiyong Ni
Guangdong Province Key Laboratory of Rare earth development and Application, Guangdong Research Institute of Rare Metals, Guangdong Academy of Sciences, Guangzhou 510651, China

Lei Chen
Intelligent manufacturing institute of Hefei University of Technology, Hefei 230051, China

This statement is signed by all the authors to indicate agreement that the above information is true and correct (*a photocopy of this form may be used if there are more than 10 authors*):

Author's name (typed)	Author's signature	Date
Liangrui He	Liangrui He.	2018. 8. 30
Mi Fei	Mi Fei	2018.8.30
Jie Chen	Jie Chen	2018.8.30
Yunfei Tian	Yunfei Tian	2018. 8. 30.
Yang Jiang	Yang Jiang	2018. 8. 30
Yang Huang	Yang Huang	2018. 8. 30
Kai Xu	Kai Xu	2018.8.30
Juntao Hu	Juntao Hu	2018. 8. 30
Zhi Zhao	Zhi Zhao	2018.8.30
Qiuhong Zhang	Qiuhong Zhang	2018. 8. 30.
Haiyong Ni	Haiyong Ni	2018. 8. 30.
Lei Chen	Lei Chen	2018. 8. 30.
